# 1154. Impact of Vaccination Protocol Implementation and Splenectomized Patient Management in a Specialized Clinic on Incidence of Encapsulated Bacterial Infections: a Retrospective Cohort Study in Quebec, Canada

**DOI:** 10.1093/ofid/ofad500.994

**Published:** 2023-11-27

**Authors:** Émilie Croft, Florence Gingras-Lessard, Jean Berchmans Musonera, Alex Carignan

**Affiliations:** Université de Sherbrooke, Sherbrooke, Quebec, Canada; Hopital Pierre Boucher, Longueuil, Quebec, Canada; Université de Sherbeooke, Sherbrooke, Quebec, Canada; Universite de Sherbrooke, Sherbrooke, QC, Canada

## Abstract

**Background:**

Splenectomy weakens immunity against encapsulated bacteria and is associated with increased overwhelming post-splenectomy infection rates. The impact of recommended vaccines for these bacterial infections has not been studied in Quebec. This study aims to i) assess changes in the incidence of encapsulated bacterial infections in patients who underwent splenectomy at *Centre Hospitalier Universitaire de Sherbrooke (CHUS)* from 1990 to 2019; ii) Investigate any changes in vaccine coverage following the implementation of: 1) a peri-splenectomy vaccination protocol in 2005 and 2) the creation of a clinic dedicated to splenectomized patients in 2015.

**Methods:**

This retrospective cohort study selected splenectomy cases performed between 1990 and 2019 at *CHUS*. Bacterial infections (*S. pneumoniae*, *H. influenzae*, and *N. meningitidis*) were identified from electronic files of microbiology laboratories. Temporal trends were assessed over three periods according to the years in which pneumococcal polysaccharide and conjugate vaccines were recommended (1990–1998; 1999–2012; 2013–2019). The primary outcome was occurrence of vaccine-preventable infection within 5 years of splenectomy.

**Results:**

The study included 544 splenectomized patients. The proportion of patients aged ≥65 years significantly increased over time [1990–1998: 30%; 1999–2012: 39%; 2013–2019: 44%; p=0.03]. *S. pneumoniae* [1990–1998: 6%; 1999–2012: 3%; 2013–2019: 0%; p=0.007] and *H. influenzae* infections significantly decreased [1990–1998: 11%; 1999–2012: 6%; 2013–2019: 0%; p=0.007]. Vaccination protocol implementation was associated with increase in the number of adequately vaccinated patients (Hib and PPV) in the 6 months after splenectomy [pre-protocol: 21%; post-protocol: 66%; p< 0.0001]. A significant difference between vaccination coverage (Hib, VPP, and VPC) in the 6 months after the splenectomy of patients seen in the immunization clinic (49%) and those not referred (8%) was observed (p< 0.0001).

**Conclusion:**

Marked decrease in post-splenectomy infection rates was observed in our population over 30 years, despite aging. Vaccination protocol implementation and splenectomized patient management in a specialized clinic can improve vaccination coverage.

**Disclosures:**

**Alex Carignan, MD, MSc**, GSK: Advisor/Consultant|GSK: Grant/Research Support|GSK: Honoraria|Moderna: Advisor/Consultant|Moderna: Honoraria|Orimed Pharma: Advisor/Consultant|Pfizer: Advisor/Consultant|Pfizer: Grant/Research Support|Pfizer: Honoraria

